# Determination of antibiotic resistance using three phenotypic methods in *Campylobacter coli* strains isolated from commercial chicken meat in Lima, Peru

**DOI:** 10.17843/rpmesp.2025.422.14330

**Published:** 2025-06-09

**Authors:** Kiara N. Cáceres-Bautista, Jorge L. Arroyo-Acevedo, Hugo J. Justil-Guerrero, Johnny A. Tinco-Jayo, Edwin C. Enciso-Roca, Enrique J. Aguilar-Felices, Miguel A. Rojas-Montes, Diego Diaz-Coahila, César A. Lázaro-de la Torre

**Affiliations:** 1 Laboratory of Veterinary Pharmacology and Toxicology, Faculty of Veterinary Medicine, National University of San Marcos. Lima, Peru. National University of San Marcos Laboratory of Veterinary Pharmacology and Toxicology Faculty of Veterinary Medicine National University of San Marcos Lima Peru; 2 Pharmacology Laboratory, Faculty of Medicine, National University of San Marcos, Lima, Peru. National University of San Marcos Pharmacology Laboratory Faculty of Medicine National University of San Marcos Lima Peru; 3 Academic Department of Human Medicine, Faculty of Health Sciences, National University of San Cristóbal de Huamanga, Ayacucho, Peru. National University of San Cristóbal de Huamanga Academic Department of Human Medicine Faculty of Health Sciences National University of San Cristóbal de Huamanga Ayacucho Peru; 4 Veterinary Immunology Laboratory, Faculty of Veterinary Medicine, National University of San Marcos. Lima, Peru. National University of San Marcos Veterinary Immunology Laboratory Faculty of Veterinary Medicine National University of San Marcos Lima Peru

**Keywords:** Antibiotic Resistance, Campylobacter coli, chicken, meat, Minimum Inhibitory Concentration

## Abstract

**Objectives.:**

To determine the resistance and minimum inhibitory concentration (MIC) of erythromycin, azithromycin, ciprofloxacin, and tetracycline in *Campylobacter coli* strains isolated from chicken carcasses sold in Lima, Peru.

**Materials and methods.:**

Cryopreserved strains of *C. coli* (n=106) were reactivated and the concordance (Kappa coefficient) of the resistance and MIC results between the disk diffusion (DD), E-test (ET), and microdilution plate (MDP) tests was evaluated.

**Results.:**

Ninety-seven strains were reactivated, of which 94 to 100% were resistant to ciprofloxacin, erythromycin, and tetracycline, while only 58% were resistant to azithromycin in the DD test. The ET and MDP tests showed 78 to 100% of resistant strains, with azithromycin presenting the lowest percentage of resistance. More than 70% of strains were resistant to at least three antibiotics in all three tests. In addition, 50%, 69%, and 100% of strains had a MIC ≥ 32 μg/mL for ciprofloxacin, azithromycin, and tetracycline/erythromycin, respectively.

**Conclusions.:**

*C. coli* strains from chicken carcasses had a high percentage of multidrug resistance. The concordance between the three tests was almost perfect, but the ET strips showed maximum concentrations that are insufficient for the MIC in these strains. It is recommended to perform resistance and MIC testing using the MDP, as it allows for a wider range of antibiotic concentrations to be used.

## INTRODUCTION

*Campylobacter spp*. is a Gram-negative bacterium that affects humans, causing short-term gastrointestinal disorders (campylobacteriosis). This process can be complicated in children, the elderly, and immunocompromised individuals, and the use of antibiotics such as fluoroquinolones, macrolides, and tetracyclines is recommended. *Campylobacter jejuni* and *Campylobacter coli* are the most common species and can be found in the intestines of broiler chickens as asymptomatic hosts. In Peru, chicken meat is the most consumed protein food (47.33 kg/person) and comes from slaughterhouses with significant sanitary deficiencies, which is a risk factor for the presence of this bacterium ^1^.

Another factor that makes *Campylobacter* spp. a bacterium of public health importance is its antibiotic resistance. The increase in resistance worldwide is making it difficult to control and monitor infections that were easily treatable decades ago, often due to self-medication ^2^. From a veterinary perspective, the use of antimicrobials during broiler chicken rearing as growth promoters or unregulated prophylactic agents is also a possible cause of resistance ^3^. In Peru, there are reports of *C. coli* strains from human clinical samples with high resistance to ciprofloxacin, nalidixic acid, tetracycline, and erythromycin, mainly associated with the *gyrA*, *aph(3´)-IIIa, tetO*, and 23S rRNA genes, respectively ^4^. The presence of the *cmeA* and *cmeB* genes, which encode efflux pumps that confer high resistance to macrolides and fluoroquinolones, has also been reported in *C. coli* strains isolated from chickens and children ^5^.

Determining resistance in bacteria related to primary human consumption products is a public health priority. In this regard, the minimum inhibitory concentration (MIC) of different antibiotics used in human and veterinary cases is of utmost importance for developing strategies and epidemiological surveillance programs. Among the phenotypic antimicrobial susceptibility methods for determining MIC are the E-test (ET), a method similar to disk diffusion (DD), which evaluates the concentration that inhibits bacterial growth with the formation of a halo around the gradient of the strip with decreasing concentrations; and the microdilution plate method (MDP), which uses microvolumes with serial dilutions to determine, like ET, the MIC with turbidity in the well ^6^^,^^7^. This study aimed to determine the minimum inhibitory concentration (MIC) of erythromycin, azithromycin, ciprofloxacin, and tetracycline in *Campylobacter coli* strains isolated from chicken meat in markets and supermarkets in Lima, Peru, using three phenotypic antimicrobial susceptibility methods.

**KEY MESSAGES**

Motivation for the study. *Campylobacter coli*, a bacterium that causes gastroenteritis in humans through the consumption of contaminated chicken meat, has shown an increase in antibiotic resistance worldwide. In Peru, information on this is scarce, so we proposed to determine resistance and minimum inhibitory concentration (MIC) using three phenotypic methods.

Main findings. In all methods, more than 70% of strains were multidrug resistant with a MIC ≥32 μg/mL, with plate microdilution being the most efficient method.

Implications. *C. coli* strains from chicken carcasses had a high percentage of multidrug resistance. Continuous monitoring with a multisectoral approach encompassing human, animal, and environmental health is necessary.

## MATERIALS AND METHODS

### Obtaining biological samples

The research was carried out at the Laboratory of Veterinary Pharmacology and Toxicology of the Faculty of Veterinary Medicine of the National University of San Marcos (Lima, Peru) between February and June 2023. A total of 106 strains of *Campylobacter coli* cryopreserved in BHI and glycerol (80:20, v/v) at -20 °C were used, characterized by genus and species through biochemical and molecular tests. These strains were collected from previous studies between 2020 and 2022 and were isolated from muscle cuts (leg with joint) and skin (from the peri-cloacal area) of chicken carcasses from markets (n=54) and supermarkets (n=52) in the districts of Independencia, La Molina, San Borja, San Martín de Porres, Santa Anita, Santiago de Surco, and Surquillo in Metropolitan Lima. Our study did not involve humans or live animals. The evaluated strains came from chicken carcasses intended for human consumption; therefore, the study did not require approval from an ethics committee.

### Reactivation of *Campylobacter coli* strains and quality controls

The sealed, intact, and coded vials were thawed in a water bath. Their contents were placed in test tubes with 3 mL of BHI culture broth enriched with 5% defibrinated sheep blood. The samples were placed in an incubator (DHP-9162, BluePard, China) at 42 °C for 36 to 48 hours under microaerophilic conditions generated by a Campygen sachet (Thermo ScientificTM OXOID™, United Kingdom). After this time, 100 µL was removed from the tube and placed in Petri dishes with mCCD agar (Thermo ScientificTM OXOID™, United Kingdom), and exhaustion plating was performed. They were incubated at 42 °C for 48 hours. After this period, the colonies were evaluated macroscopically, with those that were grayish and flat being compatible with *Campylobacter* spp., while spiral bacilli with movement were observed with the aid of a metallic sheen and under an optical microscope (Leica, United Kingdom) at 100x magnification. For the evaluation and validation of the test results, *Campylobacter jejuni* ATCC® 33560, *Campylobacter coli* ATCC® 43478, and *Staphylococcus aureus* ATCC® 29213 strains in Kwik-StickTM swab form (Microbiologics, Inc., United States), following the recommendations of the Clinical and Laboratory Standards Institute (CLSI) ^8^. For activation, the ATCC strains of *Campylobacter* were reactivated by streaking the contents of the swab on 5% blood agar at 37 °C for 48 hours under microaerophilic conditions. On the other hand, the ATTC strain of *Staphylococcus aureus* was seeded on plate count agar at 35 °C for 48 hours under aerobic conditions. From these colonies, the control solutions used in the phenotypic antimicrobial susceptibility methods were prepared.

### Disk diffusion method (DD)

From fresh colonies of *C. coli* field strains obtained from mCCD agar, a 0.5 McFarland scale solution (Liofilchem, Italy) was prepared by streaking on Müller-Hinton agar plates (Thermo ScientificTM OXOID™, United Kingdom) enriched with 5% defibrinated sheep blood. Antibiotic discs (Oxoid™, UK) containing 15 µg erythromycin (ERT), 15 µg azithromycin (AZT), 30 µg tetracycline (TET), and 5 µg ciprofloxacin (CIP) were then placed on the plates. The plates were incubated under microaerophilic conditions at 42 °C for 48 hours. The inhibition halos were then measured with a ruler and classified as sensitive (S), intermediate (I), or resistant (R) according to CLSI guidelines ^8^. For azithromycin and erythromycin, S ≥ 16 mm, I = 13-15 mm, and R ≤ 12 mm; for ciprofloxacin, S ≥ 24 mm, I = 21-23 mm, and R ≤ 20 mm; and for tetracycline, S ≥ 26 mm, I = 23-25 mm, and R ≤ 22 mm. We used ATCC strains of *C. jejuni* and *C. coli* ATCC strains, which were subjected to the same procedures described above for field strains; while for the *Staphylococcus aureus* ATCC strain, the 0.5 solution on the McFarland scale was seeded on Müller-Hinton agar and incubated at 35 °C for 18 hours under aerobic conditions.

### Microdilution in plate (MDP)

**Figure 1:**
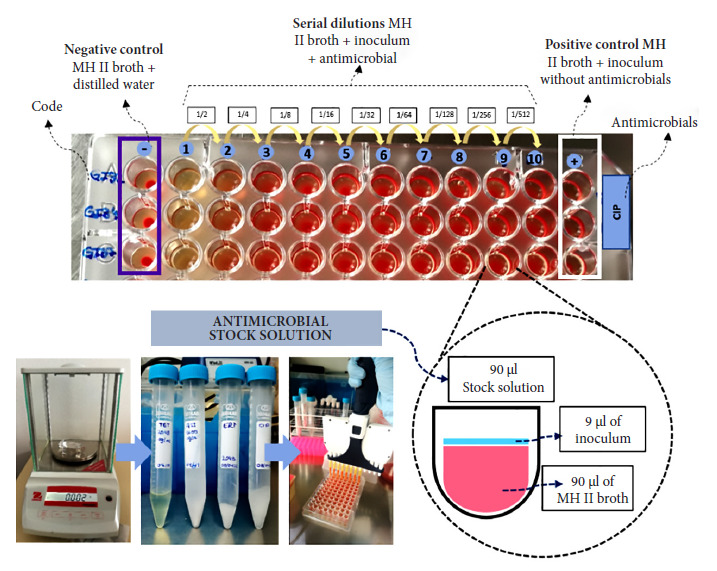
Microdilution plate wells (MDP) with serial dilutions indicating that each well contains Müller Hinton broth plus blood, the antimicrobial dilution, and the inoculum of *C. coli* strains isolated from chicken carcasses sold in markets in Metropolitan Lima in 2022 and reactivated in 2023.

To determine the MIC using this method, antibiotic standards (Sigma-Aldrich, United States) of azithromycin (1600 µg/mL), erythromycin, ciprofloxacin, and tetracycline (2048 µg/mL) were prepared. Subsequently, 90 µL of Müller-Hinton II broth (Sigma-Aldrich, United States) enriched with 5% defibrinated sheep blood was placed in the 96 wells of an oval-bottom microtiter plate (Greiner BIO-ONE, Austria), then 90 µL of the antibiotic stock solution was placed in the first well and serial dilutions of 50:50 were made in the following 10 wells. Next, 9 µL of the 0.5 McFarland scale solution of the *C. coli* field strains was added to each well (Figure 1). The plates were covered with sterile plastic film and incubated at 42 °C for 48 hours in microaerophilic medium. The MIC was evaluated by observing positive (turbidity) or negative (clarity) bacterial growth in the broth in the wells ^6^^,^^7^. The strains were classified based on the MIC value (µg/mL) according to the CLSI criteria ^8^^)^ as susceptible (S), intermediate (I), and resistant (R), where for azithromycin and erythromycin, R≥32 µg/mL, I=16 µg/mL, and S≤8 µg/mL; for ciprofloxacin, R≥4 µg/mL, I=2 µg/mL and S≤1 µg/mL; and for tetracycline, R≥16 µg/mL, I=8 µg/mL and S≤4 µg/mL. ATCC strains of *C. jejuni* and *C. coli* were used for quality control.

### E-test (ET)

This method was performed using antimicrobial strips (Liofilchem, Italy) of azithromycin (0.016-256 µg/mL), tetracycline (0.016-256 µg/mL), and ciprofloxacin (0.002-32 µg/mL), which were placed in Petri dishes with Müller-Hinton agar enriched with 5% defibrinated sheep blood and inoculated with 100 µL of a 0.5 McFarland scale solution of *Campylobacter coli*. They were then incubated under microaerophilic conditions at 42 °C for 48 hours. After this time, the parabolic or ellipsoidal inhibition zones were evaluated and the value of the strip (µg/mL) where bacterial growth inhibition began in the agar was reported. This test used the same quality controls as the MDP method and could only be performed on 88 field strains of *C. coli*, which were selected at random.

### Data analysis

The data were stored, organized, and analyzed in tables using Microsoft® Excel® for Microsoft 365 (Version 2013) on Windows. Descriptive statistics were applied using proportions. Resistance to three or more antimicrobials from different pharmacological families (9) was required to be identified as multidrug resistant; that is, resistance to the macrolide, fluoroquinolone, and tetracycline families. To analyze the degree of concordance between the results obtained using the different methods, the Kappa coefficient was calculated, with agreement values categorized as: light (0.00-0.20), fair (0.21-0.40), moderate (0.41-0.60), substantial (0.61-0.80), and almost perfect (0.81-1.00) using the online program GraphPad QuickCalcs (GraphPad Software, Dotmatics, San Diego, CA).

## RESULTS

### Antimicrobial resistance assessment

**Figure 2:**
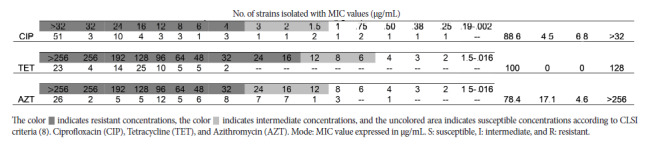
Results and classification of susceptible (S), intermediate (I), and resistant (R) for *C. coli* strains (n=88) isolated from chicken carcasses sold in markets and supermarkets in Metropolitan Lima between 2020 and 2022 and reactivated in 2023 for the E-test.

**Figure 3:**
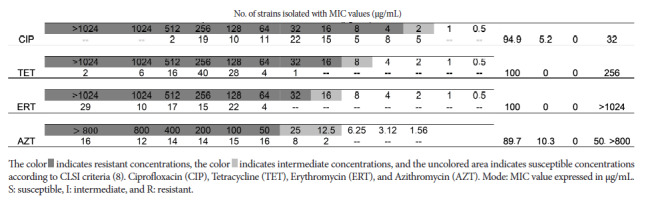
Results and classification of susceptible (S), intermediate (I), and resistant (R) for *C. coli* strains (n=97) isolated from commercial chicken carcasses sold in markets and supermarkets in Metropolitan Lima between 2020 and 2022 and reactivated in 2023 for plate microdilution testing.

A total of 97/106 (91.5%) *C. coli* strains were reactivated, and we assessed resistance and minimum inhibitory concentration. With regard to antimicrobial susceptibility testing using the DD test, of the 97 viable strains, resistance ranging from 94.9% to 100% was found for ciprofloxacin, erythromycin, and tetracycline, while for azithromycin it was 58.8% (Table 1). With regard to ET, of the total evaluated strains, 88.6% (78/88), 100% (88/88), and 78.4% (69/88) were resistant to ciprofloxacin, tetracycline, and azithromycin strips, respectively (Figure 2). The results of the MDP test (Figure 3) showed that for ciprofloxacin, 94.9% (92/97) of the evaluated strains were resistant, only 5.2% (5/97) of the strains were considered intermediate, and no strains were sensitive. For tetracycline and erythromycin, 100% (97/97) resistance was found, while 89.7% (87/97) resistant to azithromycin.

**Table 1:** Results of the disk diffusion test for all *C. coli* strains (n=97) isolated from chicken carcasses sold in markets and supermarkets in Metropolitan Lima between 2020 and 2022, reactivated in 2023, and classified according to Clinical and Laboratory Standards Institute criteria.

Antibiotic	Sensitive (%)	Intermediate (%)	Resistant (%)
Ciprofloxacin (5 µg)	3/97 (3.1%)	2/97 (2.1%)	92/97 (94.9%)
Erythromycin (15 µg)	1/97 (1.0%)	1/97 (1.0%)	95/97 (97.9%)
Azithromycin (15 µg)	12/97 (12.4%)	28/97 (28.9%)	57/97 (58.8%)
Tetracycline (30 µg).	0/97 (0%)	0/97 (0%)	97/97 (100%)

### Determination of minimum inhibitory concentration

Regarding MIC evaluated by ET, 58.0% (51/88) of strains for ciprofloxacin (>32 µg/mL); 26.1% (23/88) for tetracycline (>256 µg/mL) and 29.6% (26/88) for azithromycin (>256 µG/mL) exceeded the highest value on the strip used (Table 2). In the case of MIC by MDP, for ciprofloxacin, the strains ranged between 2 and 512 µg/mL, with 22.7% (22/97) of the strains presenting 32 µg/mL and the highest value being 512 µg/mL, but only for 2.1% (2/97) of the strains. For tetracycline and erythromycin, a MIC of 256 µg/mL was found in 41.2% (40/97) of the strains, with the highest value above 1024 µg/mL for both antimicrobials; while for azithromycin, 16.5% (16/97) of strains had MICs of 50 µg/mL and above 800 µg/mL.

**Table 2:** Number of *C. coli* strains isolated from chicken carcasses sold in markets and supermarkets in Metropolitan Lima between 2020 and 2022 and reactivated in 2023 with resistance to 1 or 4 antibiotics in the disk diffusion test (DD), E-test (ET), and microdilution plate test (MDP).

Antibiotics	DD = 97		ET = 88		MDP = 97	
	No. strains	(%)	No. strains	(%)	No. strains	(%)
TET	0	0	4	(4.6)	0	0
TET-ERT	3	(3.1)	--	--	0	0
TET-CIP	2	(2.1)	15	(17.1)	0	0
TET-AZT	0	0	6	(6.8)	0	0
TET-ERT-CIP	35	(36.1)	--	--	10	(10.3)
TET-ERT-AZT	2	(2.1)	--	--	5	(5.2)
TET- CIP-AZT	0	0	63	(71.6)	0	0
TET-CIP-AZT-ERT	55	(56.7)	--	--	82	(84.5)

Ciprofloxacin (CIP), tetracycline (TET), erythromycin (ERT), azithromycin (AZT). (--) indicates that it was not considered because ERT was not evaluated for E-test.

### Evaluation of concordance between phenotypic susceptibility methods

All three methods showed 100% of strains resistant to tetracycline. On the other hand, for ciprofloxacin, the DD and MDP methods showed 94.9% of resistant strains, unlike the ET, where only 84.6% were resistant. In the case of erythromycin, the DD method identified 98.0% of resistant strains, while the MDP method found 100% of strains to be resistant. However, azithromycin showed greater variation in results between methods, with resistance rates of 58.8%, 78.4%, and 98.0% for DD, ET, and MDP, respectively. The Kappa coefficient (κ) indicates substantial concordance (κ = 0.75) between the results of the DD and ET methods, while it was almost perfect (κ = 0.84) between the results of the DD and MDP methods and the results of the ET and MDP methods (Supplementary Material).

### Assessment of multidrug resistance

 When evaluating all strains, 84.5% (82/97) showed resistance to four antibiotics using the MDP method, while 56.7% (55/97) did so using the DD method. In addition, 71.6% (63/88) of strains showed resistance to three antibiotics using the ET method (Table 2).

## DISCUSSION

Our results showed that a high percentage of strains were resistant to ciprofloxacin with the three evaluated methods. According to Kouglenou *et al*. ^10^, resistance values for ciprofloxacin in *C. coli* strains isolated from chicken meat vary between 87 and 93.8%, which is related to the use of subtherapeutic doses of antimicrobials during chicken rearing. In contrast, Hadi *et al*. ^11^^)^ reported only 15% resistance for *C. coli* strains isolated from poultry, which they associated with the restriction of fluoroquinolones from poultry farming in the sampled area. Something similar was found for erythromycin, where more than 97% of the strains were resistant, although the ET test was not performed. This was similar to the findings by Kouglenou *et al*. ^10^^)^ and Hadi *et al*. ^11^, who found more than 95% resistance in *C. coli* strains isolated from chicken meat, possibly due to poor hygiene in the handling of the processing plant, which led to contamination, and the use of these macrolides in the poultry industry.

Tetracycline showed 100% resistance in *C. coli* samples for all three methods; resistance levels between 71.4% and 97.4% have been reported for this antibiotic in strains from chicken carcasses, suggesting that this is due to poor meat handling and backyard chicken farming ^10^^,^^12^. In contrast, Gimenez *et al*. ^13^^)^ found no resistance to erythromycin or tetracycline, but 22% of strains were resistant to ciprofloxacin. These low percentages can be attributed to adequate health management and the remote geographical location of the villages, which facilitated compliance with biosafety protocols. Another interesting finding was that only ciprofloxacin/erythromycin and azithromycin were sensitive to less than 4% and 12% of the strains, respectively. These results indicate that these groups of antibiotics would not be as effective against field strains of *C. coli* and that other antibiotic alternatives should be explored.

Gunasekaran *et al*. ^14^ found resistance rates above 60% for tetracycline, erythromycin, and azithromycin, and 21.5% for ciprofloxacin in samples of cecal mucosa from chickens. Lee *et al*. ^15^ obtained 91.1% resistance to ciprofloxacin, 71.1% to tetracycline, and 4.4% to erythromycin in strains isolated from chicken carcasses, while Lim *et al*. ^16^ reported 100% resistance to azithromycin for *C. coli* isolated from chicken meat, associating this with the use of fluoroquinolones and macrolides as routine and preventive treatments in poultry production. Pergola *et al* ^. (^^17^ and Wieczorek *et al*. ^18^ evaluated strains isolated from broiler chickens and reported resistance levels between 70% and 96.1% for ciprofloxacin and between 57.9% and 70% for tetracycline, while for erythromycin, resistance was reported between 0.6% and 30%. These results are similar to those found in this study. These authors suggest that resistance is due to the increased sale and use of fluoroquinolones in poultry production for the treatment of other diseases such as mycoplasmosis and clostridiosis.

Although resistance genes were not evaluated, it is very likely that they were present in *C. coli* strains and confer antimicrobial resistance. In the case of fluoroquinolones, the main mechanism conferring resistance is mutation of the GyrA gene, specifically in threonine at codon 86 to isoleucine (Thr86Ile), which maintains the functional capacity of DNA gyrase ^19^. In the case of macrolides, the *erm*B gene, responsible for ribosomal methylation, has been recognized as an emerging mechanism of antimicrobial resistance worldwide; likewise, specific mutations at the level of the ribosomal proteins L4 and L22 also confer resistance to macrolides ^20^. In the case of tetracyclines, the most important resistance gene is *tet*O, which protects the ribosome by preventing the antibiotic from binding and acting ^21^.

The MIC results for ciprofloxacin partially coincide with other studies that found MIC values for ciprofloxacin between 32 and 64 μg/mL for *C. coli* strains isolated from environmental swabs, feces, meat, and water in chicken slaughterhouses ^22^^,^^23^. In contrast, low MIC values between 4 and 16 µg/mL have also been reported in strains isolated from chicken cloacae and carcasses ^24^. The results for tetracycline and erythromycin were higher than those found in other studies that reported MIC values between 64 and 128 µg/mL for tetracycline and erythromycin in *Campylobacter* spp. strains isolated from chickens ^23^^-^^25^. Sadeghi *et al*. ^26^ determined a MIC of 25 μg/mL for tetracycline and ciprofloxacin, values 5 to 6 dilutions below those found by our study. They attribute this low MIC to the use of antibiotics only as a preventive measure in chicken farming. In Peru, Quino *et al*. ^4^ reported resistance rates between 52% and 100% with a MIC for erythromycin and tetracycline >128 µg/mL in *C. coli* strains isolated from human and chicken samples. The MIC for azithromycin found by our study was higher than that found by Hull *et al*. ^25^, who reported a resistance of 28% and MIC values between 0.03 and 0.12 µg/mL in strains isolated from chicken carcasses in the United States.

Our study showed a high percentage of *C. coli* strains isolated from chicken meat in Metropolitan Lima with antimicrobial resistance. This could be related to the management of birds during rearing, in which antibiotics are used as growth promoters, treatments are administered at inappropriate doses, and expired drugs are used, among other factors ^27^^,^^28^. This condition of resistant bacteria is dangerous to public health, as they can be transmitted to humans through the handling of contaminated meat or cross-contamination ^2^. Another risk is the use of the same groups of antibiotics in animals and humans. Ciprofloxacin is used to treat campylobacteriosis in humans, while enrofloxacin is the antibiotic of choice in several health programs for chicken farming. The same is happening with other groups of antibiotics that are available for use in both animals and humans ^24^. It is clear that broiler chickens are considered the primary source of human campylobacteriosis and that, despite the fact that poultry companies have control systems in place to ensure the sanitary quality of chicken meat, the total elimination of this microorganism from the food chain is complicated, especially due to raw meat handling practices or insufficient cooking by the end consumer ^29^^,^^30^.

In Peru, evidence shows that the risk of gastrointestinal diseases in children caused by *Campylobacter* spp. increases when they live with poultry (backyard farming). Therefore, containment measures such as pens and health education are recommended to reduce exposure ^31^. This is a public health problem, not only because it involves the transmission of bacteria, but also because it transmits the mechanisms of antibiotic resistance that they possess. Pollet *et al*. ^32^ reported an increase in resistance to ciprofloxacin, azithromycin, and erythromycin in strains of *C. jejuni* and *C. coli* isolated from the feces of patients with gastrointestinal problems in hospitals in Lima, Cusco, and Iquitos between 2001 and 2010. These authors suggest that the increase in resistance may be attributed to the frequent prescription of these antibiotics for infectious diseases or to self-medication; however, they also highlight that the use of macrolides and fluoroquinolones in the animal industry, especially in poultry farming, may be partly responsible for the resistance. On the other hand, Schiaffino *et al*. ^33^ reported a high incidence of quinolone- and macrolide-resistant *Campylobacter* strains in the feces of children under 2 years of age in communities on the outskirts of the city of Iquitos. They suggest that these bacteria could reach humans through domestic poultry farming and slaughter, as well as the lack of regulation on antibiotic use in poultry production.

Although the three antimicrobial susceptibility methods showed almost perfect concordance, it is important to note that DD only evaluated resistance and did not determine MIC, unlike ET and MDP. On the other hand, it is clear that the maximum antibiotic concentrations in the ET strips are not adequate for determining MIC in these strains. For this reason, the use of MDP, despite being a little more laborious, is a suitable alternative as it allows the necessary concentrations to be prepared in-house, thus providing greater versatility depending on the requirements of the strains to be evaluated. According to Paravissi *et al*. ^24^, comparing evaluation methods is challenging as there is considerable variation in procedures, parameters, and interpretation of results. Al-Natour *et al*. ^34^ evaluated the sensitivity of *C. jejuni* strains isolated from chicken feces using MDP and DD and determined that although both methodologies yielded consistent results, DD is flexible, convenient, and less laborious and could be used as an initial rapid test. On the other hand, Azrad *et al*. ^35^ found excellent concordance between ET and MDP in the evaluation of erythromycin, ciprofloxacin, and tetracycline resistance in *C. jejuni* and *C. coli* strains, highlighting that MDP has the advantage of being an automated methodology that would reduce operator bias. Lazou and Chaintoutis ^36^ found substantial and almost perfect concordance between DD and MDP for the resistance results of streptomycin, tetracycline, and nalidixic acid in strains of *C. jejuni* and *C. coli* isolated from sheep and goat meat; despite this, the authors emphasize that MDP is a quantitative method, which is valuable for clinicians when deciding on an appropriate treatment, unlike DD, which only generates qualitative data.

Resistance to three or up to twelve antimicrobials from different pharmacological families is considered to be multidrug resistance (MDR) according to Jiménez *et al*. ^9^. Our results show that more than 70% of strains were resistant to at least three antibiotics. These percentages are high compared to other authors who reported between 12.5% and 43.9% ^37^^-^^39^. Some countries have implemented corrective and preventive measures for multidrug resistance, such as the use of antibiotic-free feed for production animals ^40^. In contrast, Peru does not have an integrated resistance monitoring program for *Campylobacter*, despite the fact that in South America there are data confirming human infections and the severity of campylobacteriosis transmitted by food such as chicken meat is known, yet prevention and control measures are still scarce. Surveillance programs will help identify the problem and its severity, enabling decisions to be made and measures to be implemented ^28^. In addition, our results should also be addressed from a “One Health” perspective, since the presence of multidrug-resistant strains of *C. coli* in chicken meat poses a risk of this microorganism spreading and affecting not only humans but also pets (dogs, cats), wild animals (birds), and even insects and water, which act as disseminators; this could accelerate the horizontal transmission of *Campylobacter* resistance genes to other bacterial groups such as *Enterococcus*, *Staphylococcus*, and *Streptococcus*^41^.

One of the main limitations of this study is that only 88 strains could be evaluated for the ET test due to limitations in the number of antibiotic strips available. Furthermore, erythromycin could not be evaluated because these strips are not commercially available. Furthermore, ET is limited because the maximum concentrations of the strips were insufficient for some of the evaluated strains. In addition, DD only provides qualitative data, but it can be useful as a routine or screening method.

In conclusion, *C. coli* strains from chicken carcasses showed a high percentage of resistance to at least three antimicrobials, while for more than 50% of the strains, the MIC was greater than 32 μg/mL for ciprofloxacin, azithromycin, tetracycline, and erythromycin. Although the three methods were consistent in the evaluation of antibacterial resistance, MDP provides quantitative data and allows the used concentrations to be modified according to the sensitivity of the strains, characteristics that are advantageous over the other two methods. Since *C. coli* is a pathogenic bacterium that can be transmitted through food of animal origin, continuous monitoring with a multisectoral approach is necessary, covering human, animal, and environmental health, not only for its presence but also for antimicrobial resistance, determining the MIC, and, above all, performing an evaluation with the help of molecular biology of the resistance genes found in these strains.
