# Supplementary material for: Determination of antibiotic resistance using three phenotypic methods in Campylobacter coli strains isolated from commercial chicken meat in Lima, Peru
Source: Rev Peru Med Exp Salud Publica. 2025 Jun 9;42(2):147–55. doi: 10.17843/rpmesp.2025.422.14330 (PMC12380429; doi:10.17843/rpmesp.2025.422.14330)
Supplement: Supplementary material. — Available in the electronic version of the RPMESP. [file rpmesp-42-02-14330-s001.docx]

**Material suplementario**

Anexo 1. Resultados de la concordancia entre las pruebas de difusión en disco (DD) y microdilución en placa (MDP) de las cepas de *C. coli* aisladas de Carcasas de pollo comercializadas en mercados y supermercados de Lima Metropolitana entre el 2020 al 2022 que fueron reactivadas en el 2023 y categorizadas como sensible (S), intermedio (I) y resistente (R).

|  |  |  | MDP | | | | | | | | | | | |  |
| --- | --- | --- | --- | --- | --- | --- | --- | --- | --- | --- | --- | --- | --- | --- | --- |
|  |  |  | CIP | | | TET | | | ERT | | | AZT | | |  |
|  |  |  | R | S | I | R | S | I | R | S | I | R | S | I | Total |
| DD | CIP | R | 89 | 0 | 3 | 0 | 0 | 0 | 0 | 0 | 0 | 0 | 0 | 0 | 92 |
|  |  | S | 2 | 0 | 1 | 0 | 0 | 0 | 0 | 0 | 0 | 0 | 0 | 0 | 3 |
|  |  | I | 2 | 0 | 0 | 0 | 0 | 0 | 0 | 0 | 0 | 0 | 0 | 0 | 2 |
|  | TET | R | 0 | 0 | 0 | 97 | 0 | 0 | 0 | 0 | 0 | 0 | 0 | 0 | 97 |
|  |  | S | 0 | 0 | 0 | 0 | 0 | 0 | 0 | 0 | 0 | 0 | 0 | 0 | 0 |
|  |  | I | 0 | 0 | 0 | 0 | 0 | 0 | 0 | 0 | 0 | 0 | 0 | 0 | 0 |
|  | ERT | R | 0 | 0 | 0 | 0 | 0 | 0 | 95 | 0 | 0 | 0 | 0 | 0 | 95 |
|  |  | S | 0 | 0 | 0 | 0 | 0 | 0 | 1 | 0 | 0 | 0 | 0 | 0 | 1 |
|  |  | I | 0 | 0 | 0 | 0 | 0 | 0 | 1 | 0 | 0 | 0 | 0 | 0 | 1 |
|  | AZT | R | 0 | 0 | 0 | 0 | 0 | 0 | 0 | 0 | 0 | 54 | 0 | 3 | 57 |
|  |  | S | 0 | 0 | 0 | 0 | 0 | 0 | 0 | 0 | 0 | 10 | 0 | 2 | 12 |
|  |  | I | 0 | 0 | 0 | 0 | 0 | 0 | 0 | 0 | 0 | 23 | 0 | 5 | 28 |
|  | Total |  | 93 | 0 | 4 | 97 | 0 | 0 | 97 | 0 | 0 | 87 | 0 | 10 | 388 |

Cuantificación de la concordancia por el Coeficiente de Kappa (κ)

- Número de observaciones concordantes: 340 (87,6% de las observaciones)
- Número de observaciones concordantes esperadas al azar: 83,6 (21,5% de las observaciones)
- Kappa= 0,842
- Error estándar de kappa = 0,020
- Intervalo de confianza al 95%: De 0,802 a 0,882
- Kappa ponderado = 0,948

**Anexo 2.** Resultados de la concordancia entre las pruebas de difusión en disco (DD) y E-test (ET) de las cepas de *C. coli* aisladas de Carcasas de pollo comercializadas en mercados y supermercados de Lima Metropolitana entre el 2020 al 2022 que fueron reactivadas en el 2023 y categorizadas como sensible (S), intermedio (I) y resistente (R).

|  |  |  | DD | | | | | | | | |  |
| --- | --- | --- | --- | --- | --- | --- | --- | --- | --- | --- | --- | --- |
|  |  |  | CIP | | | TET | | | AZT | | |  |
|  |  |  | R | S | I | R | S | I | R | S | I | Total |
| ET | CIP | R | 78 | 0 | 0 | 0 | 0 | 0 | 0 | 0 | 0 | 78 |
|  |  | S | 2 | 2 | 2 | 0 | 0 | 0 | 0 | 0 | 0 | 6 |
|  |  | I | 4 | 0 | 0 | 0 | 0 | 0 | 0 | 0 | 0 | 4 |
|  | TET | R | 0 | 0 | 0 | 88 | 0 | 0 | 0 | 0 | 0 | 88 |
|  |  | S | 0 | 0 | 0 | 0 | 0 | 0 | 0 | 0 | 0 | 0 |
|  |  | I | 0 | 0 | 0 | 0 | 0 | 0 | 0 | 0 | 0 | 0 |
|  | AZT | R | 0 | 0 | 0 | 0 | 0 | 0 | 43 | 8 | 18 | 69 |
|  |  | S | 0 | 0 | 0 | 0 | 0 | 0 | 0 | 0 | 4 | 4 |
|  |  | I | 0 | 0 | 0 | 0 | 0 | 0 | 6 | 4 | 5 | 15 |
|  | Total |  | 84 | 2 | 2 | 88 | 0 | 0 | 49 | 12 | 27 | 264 |

Cuantificación de la concordancia por el Coeficiente de Kappa (κ)

- Número de observaciones concordantes: 216 (81,82% de las observaciones)
- Número de observaciones concordantes esperadas al azar: 68,8 (26,0% de las observaciones)
- Kappa= 0,754
- Error estándar de kappa = 0,029
- Intervalo de confianza al 95%: De 0,697 a 0,812
- Kappa ponderado = 0,904

**Anexo 3.** Resultados de la concordancia entre las pruebas de E-test (ET) y microdilución en placa (MDP) de las cepas de *C. coli* aisladas de Carcasas de pollo comercializadas en mercados y supermercados de Lima Metropolitana entre el 2020 al 2022 que fueron reactivadas en el 2023 y categorizadas como sensible (S), intermedio (I) y resistente (R).

|  |  |  | MDP | | | | | | | | |  |
| --- | --- | --- | --- | --- | --- | --- | --- | --- | --- | --- | --- | --- |
|  |  |  | CIP | | | TET | | | AZT | | |  |
|  |  |  | R | S | I | R | S | I | R | S | I | Total |
| ET | CIP | R | 76 | 0 | 2 | 0 | 0 | 0 | 0 | 0 | 0 | 78 |
|  |  | S | 4 | 0 | 2 | 0 | 0 | 0 | 0 | 0 | 0 | 6 |
|  |  | I | 4 | 0 | 0 | 0 | 0 | 0 | 0 | 0 | 0 | 4 |
|  | TET | R | 0 | 0 | 0 | 88 | 0 | 0 | 0 | 0 | 0 | 88 |
|  |  | S | 0 | 0 | 0 | 0 | 0 | 0 | 0 | 0 | 0 | 0 |
|  |  | I | 0 | 0 | 0 | 0 | 0 | 0 | 0 | 0 | 0 | 0 |
|  | AZT | R | 0 | 0 | 0 | 0 | 0 | 0 | 66 | 0 | 4 | 70 |
|  |  | S | 0 | 0 | 0 | 0 | 0 | 0 | 3 | 0 | 1 | 4 |
|  |  | I | 0 | 0 | 0 | 0 | 0 | 0 | 10 | 0 | 4 | 14 |
|  | Total |  | 84 | 0 | 4 | 88 | 0 | 0 | 79 | 0 | 9 | 264 |

Cuantificación de la concordancia por el Coeficiente de Kappa (κ)

- Número de observaciones concordantes: 234 (88,6% de las observaciones)
- Número de observaciones concordantes esperadas al azar: 75,6 (28,6% de las observaciones)
- Kappa= 0,841
- Error estándar de kappa = 0,026
- Intervalo de confianza al 95%: De 0,790 a 0,892
- Kappa ponderado = 0,933
