# Supplementary material for: Determination of antibiotic resistance using three phenotypic methods in Campylobacter coli strains isolated from commercial chicken meat in Lima, Peru
Source: Rev Peru Med Exp Salud Publica. 2025 Jun 9;42(2):147–55. doi: 10.17843/rpmesp.2025.422.14330 (PMC12380429; doi:10.17843/rpmesp.2025.422.14330)
Supplement: Translation

# Translation: Determinación de la resistencia a antibióticos mediante tres métodos fenotípicos en cepas de *Campylobacter coli* aisladas de carne de pollo comercializada en Lima, Perú

**Authors:** Kiara N. Cáceres-Bautista, Jorge L. Arroyo-Acevedo, Hugo J. Justil-Guerrero, Johnny A. Tinco-Jayo, Edwin C. Enciso-Roca, Enrique J. Aguilar-Felices, Miguel A. Rojas-Montes, Diego Diaz-Coahila, César A. Lázaro-de la Torre

**Affiliations:** 1 Laboratorio de Farmacología y Toxicología Veterinaria, Facultad de Medicina Veterinaria, Universidad Nacional Mayor de San Marcos. Lima, Perú.; 2 Laboratorio de Farmacología, Facultad de Medicina, Universidad Nacional Mayor de San Marcos, Lima, Perú.; 3 Departamento Académico de Medicina Humana, Facultad de Ciencias de la Salud, Universidad Nacional de San Cristóbal de Huamanga, Ayacucho, Perú.; 4 Laboratorio de Inmunología Veterinaria, Facultad de Medicina Veterinaria, Universidad Nacional Mayor de San Marcos. Lima, Perú.

**Keywords:** Resistencia a Antibióticos, Campylobacter coli, carne, pollo, Concentración Inhibitoria Mínima

## Abstract

**Objetivos.:**

Determinar la resistencia y concentración inhibitoria mínima (CIM) de eritromicina, azitromicina, ciprofloxacina y tetraciclina en cepas de *Campylobacter coli* aisladas de carcasas de pollos comercializados en Lima, Perú.

**Materiales y métodos.:**

Cepas de *C. coli* (n=106) criopreservadas se reactivaron y se evaluó la concordancia (Coeficiente Kappa) de los resultados de resistencia y CIM entre las pruebas de difusión en disco (DD), E-test (ET) y microdilución en placa (MDP).

**Resultados.:**

Se reactivaron 97 cepas de las cuales entre 94 al 100% fueron resistentes a ciprofloxacina, eritromicina y tetraciclina; mientras que solo el 58% fue a azitromicina en la prueba de DD. Las pruebas de ET y MDP evidenciaron entre 78 al 100% de cepas resistentes, siendo la azitromicina que presentó el menor porcentaje de resistencia. Más del 70% de cepas presentaron resistencia a por lo menos tres antibióticos en las tres pruebas. Además, el 50%, 69% y 100% de cepas presentaron una CIM ≥ 32 μg/mL para ciprofloxacina, azitromicina y tetraciclina/eritromicina, respectivamente.

**Conclusiones.:**

Las cepas de *C. coli* provenientes de carcasas de pollos tuvieron un alto porcentaje de multidrogorresistencia. La concordancia entre las tres pruebas fue casi perfecta, pero las tiras del ET presentaron concentraciones máximas que son insuficientes para la CIM en estas cepas. Es recomendable realizar la evaluación de la resistencia y la CIM mediante la MDP ya que permite utilizar un mayor rango de concentraciones de los antibióticos.

## INTRODUCCIÓN

*Campylobacter* spp. es una bacteria Gram negativa que afecta al hombre ocasionando desordenes gastroentéricos de corta duración (Campilobacteriosis); este proceso se puede complicar en niños, ancianos e inmunocomprometidos, siendo recomendado el uso de antibióticos como fluoroquinolonas, macrólidos y tetraciclinas. *Campylobacter jejuni* y *Campylobacter coli* son las especies más frecuentes y se pueden encontrar en el intestino de los pollos de carne como hospederos asintomáticos. En el Perú, la carne de pollo es el alimento proteico más consumido (47,33 kg/persona) la cual proviene de mataderos con notables deficiencias sanitarias, siendo esto un factor de riesgo para la presentación de esta bacteria ^1^.

Otro factor que hace al *Campylobacter* spp. una bacteria de importancia en salud pública es su característica de resistencia antibiótica. El incremento de la resistencia a nivel mundial está generando dificultad en el control y vigilancia de infecciones que en décadas atrás eran fácilmente tratables, muchas veces esto se relacionaba a la automedicación de las personas ^2^. Desde el punto de vista veterinario el uso de antimicrobianos durante la crianza de pollos de carne como promotores de crecimiento o agentes profilácticos no regulados también es una posible causa de la resistencia ^3^. En el Perú existen reportes de cepas de *C. coli* provenientes de muestras clínicas en humanos con alta resistencia a ciprofloxacina, ácido nalidíxico, tetraciclina y eritromicina asociados principalmente a genes *gyr*A, *aph(3´)-IIIa*, *tetO* y 23S rRNA respectivamente ^4^, también se ha reportado la presencia de genes *cmeA* y *cmeB* los cuales codifican las bombas de eflujo que confieren una alta resistencia a macrólidos y fluoroquinolonas en cepas *de C. coli* aisladas de pollos y de niños ^5^.

Determinar la resistencia en bacterias que estén relacionadas a productos de consumo humano primario es de prioridad en salud pública. En este sentido, la concentración inhibitoria mínima (CIM) de diferentes antibióticos aplicados en casos humanos y veterinarios es de suma importancia para plantear estrategias y desarrollar programas de vigilancia epidemiológica. Entre los métodos de sensibilidad antimicrobiana fenotípicas para determinar la CIM tenemos el E-test (ET), método similar a difusión en disco (DD), evalúa la concentración que inhibe el crecimiento bacteriano con la formación de un halo alrededor de la gradiente de la tira con concentraciones decrecientes; y el método de microdilución en placa (MDP), la cual usa micro volúmenes con diluciones seriadas que determinaran al igual que ET la CIM con turbidez en el pocillo ^6^^,^^7^. El objetivo del presente trabajo es determinar la concentración inhibitoria mínima (CIM) de eritromicina, azitromicina, ciprofloxacina y tetraciclina en cepas de *Campylobacter coli* aisladas de carne de pollos en mercados y supermercados de Lima, Perú utilizando tres métodos de sensibilidad antimicrobiana fenotípica.

**MENSAJE CLAVE**

Motivación para realizar el estudio. *Campylobacter coli*, bacteria que produce gastroenteritis en humanos por consumo de carne de pollo contaminada, ha mostrado un incremento de la resistencia antibiótica a nivel mundial. En Perú esta información es escasa, por lo que se propuso determinar la resistencia y la concentración inhibitoria mínima (CIM) con tres métodos fenotípicos.

Principales hallazgos. En todos los métodos más del 70% de cepas presentaron multidrogorresistencia con una CIM ≥32 μg/mL, siendo la microdilución en placa el método más eficiente.

Implicancias. Las cepas de *C. coli* provenientes de carcasas de pollos tuvieron un alto porcentaje de multidrogorresistencia. Es necesario un continuo monitoreo con un enfoque multisectorial que abarque la salud humana, animal y el medio ambiente.

## MATERIALES Y MÉTODOS

### Obtención de muestras biológicas

El trabajo de investigación se llevó a cabo en el Laboratorio de Farmacología y Toxicología Veterinaria de la Facultad de Medicina Veterinaria de la Universidad Nacional Mayor de San Marcos (Lima-Perú) entre los meses de febrero a junio del 2023. Se utilizaron 106 cepas de *Campylobacter coli* criopreservadas en BHI y glicerol (80:20, v/v) a -20 °C, caracterizadas en género y especie por pruebas bioquímicas y moleculares. Estas cepas fueron colectadas de trabajos previos entre los años 2020 y 2022 las cuales fueron aisladas de cortes de músculo (pierna con encuentro) y piel (de la zona peri-cloacal) de carcasas de pollo de mercados (n=54) y supermercados (n=52) de los distritos de Independencia, La Molina, San Borja, San Martín de Porres, Santa Anita, Santiago de Surco y Surquillo en Lima Metropolitana. El presente estudio no involucró humanos ni animales vivos. Las cepas evaluadas provenían de carcasas de pollos destinados a consumo humano; por consiguiente, el estudio no requirió aprobación de un comité de ética.

### Reactivación de cepas de *Campylobacter coli* y controles de calidad

Los viales cerrados, íntegros y con codificación fueron descongelados en baño María. Su contenido se colocó en tubos de ensayo con 3 mL de caldo de cultivo BHI enriquecido con sangre desfibrinada de ovino al 5%. Las muestras fueron llevadas a la incubadora (DHP-9162, **BluePard,** China) a 42 °C por 36 a 48 horas en condiciones microaerófilas generada por un sachet de Campygen (Thermo Scientific^TM^ OXOID™, Reino Unido), pasado este tiempo se procedió a retirar 100 µL del tubo y se colocó en placas Petri con agar mCCD (Thermo Scientific^TM^ OXOID™, Reino Unido) haciéndose una siembra por agotamiento. Se llevaron a incubación a 42 °C por 48 horas. Pasado este periodo las colonias fueron evaluadas macroscópicamente, siendo compatibles con *Campylobacter* spp. las de color grisáceas, planas "; mientras que bacilos espiralados con movimiento fueron observados con ayuda de un brillo metálico y bajo un microscopio óptico (Leica, Reino Unido) a 100x. Para la evaluación y validación de los resultados de las pruebas fueron considerados como controles de calidad las cepas de *Campylobacter jejuni* ATCC® 33560, *Campylobacter coli* ATCC® 43478 y *Staphylococcus aureus* ATCC® 29213 en presentación de hisopo Kwik-StickTM (Microbiologics, Inc., Estados Unidos), esto siguiendo las recomendaciones del Clinical and Laboratory Standards Institute (CLSI) ^8^. Para su activación las cepas ATCC de *Campylobacter* fueron reactivadas estriando por agotamiento el contenido del hisopo en agar sangre al 5% a 37 °C por 48 horas en condiciones de microaerófilia. Por otro lado, la cepa ATTC de *Staphylococcus aureus* fue sembrada en agar de conteo de placas a 35 °C por 48 horas en condiciones de aerobiosis. A partir de estas colonias se procedió a preparar las soluciones control usadas en los métodos de sensibilidad antimicrobiana fenotípica. 

### Método de difusión en disco (DD)

A partir de colonias frescas de las cepas de campo de *C. coli* obtenidas del agar mCCD se preparó una solución al 0,5 en la escala de McFarland (Liofilchem, Italia), para esto se procedió a hacer el sembrado por estriamiento en placas de agar Müller-Hinton (Thermo Scientific^TM^ OXOID™, Reino Unido) enriquecida con sangre desfibrinada de oveja al 5%. Seguidamente se colocaron discos antibióticos (Oxoid™, Reino Unido) de eritromicina 15 µg (ERT), azitromicina 15 µg (AZT), tetraciclina 30 µg (TET) y ciprofloxacino 5 µg (CIP). Las placas fueron incubadas en condiciones microaerófilas a 42 °C durante 48 horas. Posteriormente se midió los halos de inhibición con una regla y la clasificación de sensible (S), intermedio (I) y resistente (R) fue realizada según las indicaciones del CLSI ^8^. En el caso de azitromicina y eritromicina fue S ≥ 16 mm, I = 13-15 mm y R ≤ 12 mm; para ciprofloxacina fue S ≥ 24 mm, I = 21-23 mm y R ≤ 20 mm; y para la tetraciclina fue S ≥ 26 mm, I = 23-25 mm y R ≤ 22 mm. Para realizar el control de calidad se utilizaron las cepas ATCC de *C. jejuni,* y *C. coli*, las cuales fueron sometidas a los mismos procedimientos descritos anteriormente para las cepas de campo; mientras que para la cepa ATCC de *Staphylococcus aureus* la solución 0,5 en la escala de McFarland se sembró en agar Müller-Hinton y la incubación fue a 35 °C por 18 horas en aerobiosis.

### Microdilución en placa (MDP)

Para la determinación de la CIM por este método se prepararon estándares de antibióticos (Sigma-Aldrich, Estados Unidos) de azitromicina (1600 µg/mL), eritromicina, ciprofloxacina y tetraciclina (2048 µg/mL). Posteriormente, se colocó 90 µL de caldo Müller-Hinton II (Sigma-Aldrich, Estados Unidos) enriquecido con sangre desfibrinada de ovino al 5% en los 96 pocillos de una placa de microtitulación de fondo ovalado (Greiner BIO-ONE, Austria), después se colocó 90 µL de la solución stock de antibiótico en el primer pocillo y se realizaron diluciones seriadas 50:50 en los siguientes 10 pocillos. Luego se adicionó a cada pocillo 9 µL de la solución de 0,5 en la escala de McFarland de las cepas de campo de *C. coli* (Figura 1). Las placas fueron cubiertas con papel film estéril e incubadas a 42 °C durante 48 horas en medio microaerófilo. La CIM fue evaluada con la observación del crecimiento bacteriano positivo (turbidez) o negativo (claridad) en el caldo de los pocillos ^6^^,^^7^. Las cepas fueron clasificadas en base al valor de la CIM (µg/mL) según los criterios de sensible (S), intermedio (I) y resistente (R) del CLSI ^8^ donde para azitromicina y eritromicina fue R≥32 µg/mL, I=16 µg/mL y S≤8 µg/mL; para ciprofloxacina fue R≥4 µg/mL, I=2 µg/mL y S≤1 µg/mL; y para la tetraciclina fue R≥16 µg/mL, I=8 µg/mL y S≤4 µg/mL. Para realizar el control de calidad se utilizaron las cepas ATCC de *C. jejuni,* y *C. coli.*

**Figura 1:** Pocillos de placas de microdilución en placa (MDP) con diluciones seriadas indicando que en cada pocillo se encuentra el caldo Müller Hinton más sangre, la dilución de antimicrobiano y el inóculo de cepas de C. coli aisladas de carcasas de pollo comercializadas en mercados de Lima Metropolitana en el 2022 y reactivadas en el 2023.

### E-test (ET)

Este método fue realizado mediante tiras de antimicrobianos (Liofilchem, Italia) de azitromicina (0,016-256 µg/mL), tetraciclina (0,016-256 µg/mL), y ciprofloxacina (0,002-32 µg/mL) las cuales fueron colocadas en placas Petri con agar Müller-Hinton enriquecida con sangre desfibrinada de ovino al 5% e inoculadas con 100 µL de una solución de *Campylobacter coli* al 0,5 en la escala de McFarland. Luego fueron llevados a incubación en condiciones de microaerófilia a 42 °C durante 48 horas. Pasado este tiempo se evaluaron las zonas de inhibición de forma parabólica o elipsoide y se observó el valor de la tira (µg/mL) donde se inicia la inhibición del crecimiento bacteriano en el agar. Esta prueba utilizó los mismos controles de calidad que el método MDP y solo pudo ser realizada en 88 cepas de campo de *C. coli* las cuales fueron seleccionadas aleatoriamente.

### Análisis de datos

Los datos fueron almacenados, organizados y analizados en tablas utilizando Microsoft® Excel® para Microsoft 365 (Versión 2013) en Windows. Se aplicó estadística descriptiva haciendo uso de proporciones. Para ser identificado como multidrogorresistente debía presentar resistencia a tres o más antimicrobianos de distintas familias farmacológicas ^9^; es decir, debe presentar resistencia a la familia de macrólidos, fluoroquinolonas y tetraciclinas. Para analizar el grado de concordancia de los resultados obtenidos entre los métodos se calculó el coeficiente de Kappa siendo los valores de concordancia categorizados como: ligero (0,00-0,20), regular (0,21-0,40), moderado (0,41-0,60) sustancial (0,61-0,80) y casi perfecta (0,81-1,00) utilizando el programa online GraphPad QuickCalcs (GraphPad Software, Dotmatics, San Diego, CA).

## RESULTADOS

### Evaluación de la resistencia antimicrobiana

En total pudieron ser reactivadas 97/106 (91,5%) de cepas de *C. coli*., a partir de estas se realizó la evaluación de la resistencia y la concentración inhibitoria mínima. Con respecto a la evaluación de sensibilidad antimicrobiana evaluada con la prueba DD, de las 97 cepas viables se encontró una resistencia entre 94,9% a 100% para ciprofloxacina, eritromicina y tetraciclina; mientras que para azitromicina fue de 58,8% (Tabla 1). Con respecto al ET, en el total de las cepas evaluadas el 88,6% (78/88), 100% (88/88) y 78,4% (69/88) fueron resistentes para las tiras de ciprofloxacina, tetraciclina y azitromicina respectivamente (Figura 2). Los resultados de la prueba de MDP (Figura 3) mostraron que para ciprofloxacina el 94,9% (92/97) de cepas evaluadas fueron resistentes, solo 5,2% (5/97) de cepas fueron consideradas como intermedias y ninguna cepa fue sensible. Para tetraciclina y eritromicina se encontró un 100% (97/97) de resistencia, mientras que para azitromicina se obtuvo un 89,7% (87/97) de resistencia.

**Tabla 1:** Resultados de la prueba de difusión en disco del total de cepas de *C. coli* (n=97) aisladas de carcasas de pollo comercializadas en mercados y supermercados de Lima Metropolitana entre el 2020 al 2022 reactivadas en el 2023 y clasificadas según criterios del Clinical and Laboratory Standards Institute.

Antimicrobiano	Sensible (%)	Intermedio (%)	Resistente (%)
Ciprofloxacina (5 µg)	3/97 (3,1%)	2/97 (2,1%)	92/97 (94,9%)
Eritromicina (15 µg)	1/97 (1,0%)	1/97 (1,0%)	95/97 (97,9%)
Azitromicina (15 µg)	12/97 (12,4%)	28/97 (28,9%)	57/97 (58,8%)
Tetraciclina (30 µg).	0/97 (0%)	0/97 (0%)	97/97 (100%)

**Figura 2:** Resultados y clasificación de sensible (S), intermedio (I) y resistente (R) para las cepas de *C. coli* (n=88) aisladas de carcasas de pollo comercializadas en mercados y supermercados de Lima Metropolitana entre el 2020 al 2022 y reactivadas en el 2023 para la prueba de E-test.

**Figura 3:** Resultados y clasificación de sensible (S), intermedio (I) y resistente (R) para las cepas de *C. coli* (n=97) aisladas de Carcasas de pollo comercializadas en mercados y supermercados de Lima Metropolitana entre el 2020 al 2022 y reactivadas en el 2023 para la prueba de microdilución en placa.

### Determinación de la concentración inhibitoria mínima

En cuanto a la CIM evaluada por ET el 58,0% (51/88) de cepas para ciprofloxacina (>32 µg/mL); 26,1% (23/88) para tetraciclina (>256 µg/mL) y 29,6% (26/88) para azitromicina (>256 µg/mL) superaron el valor más alto de la tira utilizada (Tabla 2). En el caso de la CIM por MDP, para ciprofloxacina las cepas presentaron un rango entre 2 y 512 µg/mL, siendo que el 22,7% (22/97) de las cepas presentaron 32 µg/mL y el valor más alto fue 512 µg/mL pero solo para 2,1% (2/97) cepas. Para tetraciclina y eritromicina se presentó una CIM de 256 µg/mL en el 41,2% (40/97) de las cepas y siendo el valor más alto por encima de 1024 µg/mL para ambos antimicrobianos; mientras que, para azitromicina, 16,5% (16/97) de las cepas presentaron CIM de 50 µg/mL y superior a 800 µg/mL.

**Tabla 2:** Cantidad de cepas de *C. coli* aisladas de carcasas de pollo comercializadas en mercados y supermercados de Lima Metropolitana entre el 2020 al 2022 y reactivadas en el 2023 con característica de resistencia a 1 o 4 antibióticos en la prueba de difusión en disco (DD), E-test (ET) y microdilución en placa (MDP).

Antimicrobianos	DD = 97		ET = 88		MDP = 97	
	Nº Cepas	(%)	Nº Cepas	(%)	Nº Cepas	(%)
TET	0	0	4	(4,6)	0	0
TET-ERT	3	(3,1)	--	--	0	0
TET-CIP	2	(2,1)	15	(17,1)	0	0
TET-AZT	0	0	6	(6,8)	0	0
TET-ERT-CIP	35	(36,1)	--	--	10	(10,3)
TET-ERT-AZT	2	(2,1)	--	--	5	(5,2)
TET- CIP-AZT	0	0	63	(71,6)	0	0
TET-CIP-AZT-ERT	55	(56,7)	--	--	82	(84,5)

Ciprofloxacina (CIP), tetraciclina (TET), eritromicina (ERT), azitromicina (AZT). (--) indica que no se consideró porque para E-test no se evaluó ERT.

### Evaluación de la concordancia entre los métodos de sensibilidad fenotípicos

Los tres métodos evidenciaron un 100% de cepas resistentes a tetraciclina. Por otro lado, para ciprofloxacina los métodos de DD y MDP evidenciaron un 94,9% de cepas resistentes a diferencia del ET donde solo se encontraron 84,6% de resistencia. En el caso de los resultados para eritromicina el método de DD identificó un 98,0% de cepas resistentes mientras que en el método de MDP el 100% de las cepas presentaron resistencia. Sin embargo, la azitromicina tuvo mayor variación de los resultados entre los métodos presentando porcentajes de resistencia de 58,8%, 78,4% y 98,0% para DD, ET y MDP respectivamente. El coeficiente de Kappa (κ) indica una concordancia categorizada como sustancial (κ = 0,75) entre los resultados de los métodos DD y ET, mientras que fue casi perfecta (κ = 0,84) entre los resultados de los métodos DD y MDP y los resultados de los métodos ET y MDP (Material suplementario).

### Evaluación de la condición de multidrogorresistencia

Al hacer la evaluación del total de cepas el 84,5% (82/97) presentaron resistencia para 4 antibióticos con el método de MDP, mientras que un 56,7% (55/97) en el método DD; además el 71,6% (63/88) de las cepas presentaron resistencia para 3 antibióticos en el método de ET (Tabla 2).

## DISCUSIÓN

Nuestros resultados mostraron que un alto porcentaje de cepas fueron resistente para ciprofloxacina en los tres métodos evaluados. Según Kouglenou *et al.*^10^ los valores de resistencia para ciprofloxacina en cepas de *C. coli* aisladas de carne de pollo varían entre 87 a 93,8% y esto se relaciona con el uso de antimicrobianos en dosis subterapéuticas durante la crianza de los pollos. En contraposición, Hadi *et al.*^11^ muestran solo un 15% de resistencia para cepas de *C. coli* aisladas de aves, esto lo asociaron con la restricción de fluoroquinolonas en avicultura de la zona muestreada. Algo similar se observó para eritromicina donde más del 97% de las cepas fue resistente, a pesar que no fue realizada la prueba de ET. Esto fue similar a lo reportado por Kouglenou *et al.*^10^ y Hadi *et al.*^11^ quienes encontraron más de 95% de resistencia en cepas de *C. coli* aisladas en carne de pollo, posiblemente por deficiencia de higiene en el manejo del centro de beneficio que originó la contaminación y por el uso de estos macrólidos en la industria avícola.

La tetraciclina presentó un 100% de resistencia para *C. coli* en los tres métodos; niveles entre 71,4% y 97,4% de resistencia han sido reportados para este antibiótico en cepas provenientes de carcasas de pollo, sugiriendo que esto se debe al manejo deficiente de la carne y la crianza de traspatio de los pollos ^10^^,^^12^. En contraposición, Gimenez *et al.*^13^ no encontraron resistencia para eritromicina ni tetraciclina, pero si un 22% de cepas resistentes a ciprofloxacina, estos bajos porcentajes pueden ser atribuidos al adecuado manejo sanitario realizado y por la ubicación geográfica alejada de los pueblos que facilitó el cumplimiento de los protocolos de bioseguridad. Otro dato interesante fue que solo ciprofloxacina/eritromicina y azitromicina fueron sensibles para menos del 4% y 12% de las cepas respectivamente. Estos resultados indican que estos grupos de antibióticos no serían tan eficaces frente a las cepas de campo de *C. coli* y que deberían explorase otras alternativas antibióticas.

Gunasekaran *et al.*^14^ encontraron porcentajes de resistencia superiores a 60% para tetraciclina, eritromicina y azitromicina y 21,5% para ciprofloxacina en muestras de mucosa cecal de pollos. Lee *et al.*^15^ obtuvieron 91,1% de resistencia para ciprofloxacina, 71,1% para tetraciclina y 4,4% para eritromicina en cepas aisladas de carcasas de pollo, mientras que Lim *et al.*^16^ reportaron 100% de resistencia para azitromicina para *C. coli* aislada en carne de pollo, asociando esto al uso de fluoroquinolonas y macrólidos como tratamientos rutinarios y preventivos en producción aviar. Pergola *et al.*^17^ y Wieczorek *et al.*^18^, evaluaron cepas aisladas de pollos de carne y reportaron niveles de resistencia entre 70% a 96,1% para ciprofloxacina y de 57,9% a 70% para tetraciclina; mientras que para eritromicina se reportó entre 0,6 a 30%, estos resultados son cercanos a lo encontrado en este estudio. Estos autores sugieren que esta condición de resistencia es por el aumento de venta y uso de fluoroquinolonas en la producción avícola para tratamiento de otras enfermedades como la micoplasmosis y clostridiosis.

A pesar que no fue realizado una evaluación de los genes de resistencia es muy probable que estén presentes en las cepas de *C. coli* y sean los que confieren resistencia antimicrobiana. En el caso de las fluoroquinolonas, el mecanismo principal que confiere resistencia es la mutación del gen GyrA, específicamente en la treonina del codón 86 por una isoleucina (Thr86Ile) con lo cual la capacidad funcional de la DNA girasa se mantiene estable ^19^. En el caso de los macrólidos, el gen *erm*B, responsable de la metilación ribosomal, ha sido reconocido como un mecanismo emergente de resistencia antimicrobiana a nivel mundial; asimismo, mutaciones específicas a nivel de las proteínas ribosomales L4 y L22 también confieren resistencia a macrólidos ^20^. En el caso de las tetraciclinas los genes de resistencia más importante es el *tet*O el cual confieren protección al ribosoma al evitar que el antibiótico se una y actúe ^21^.

Los resultados de CIM para ciprofloxacina coinciden parcialmente con otros estudios que encontraron valores de CIM para ciprofloxacina entre 32 y 64 μg/mL para cepas de *C. coli* aisladas de hisopados ambientales, heces, carne y agua en mataderos de pollos ^22^^,^^23^. Contrariamente, también se reportan valores de CIM bajos entre 4 y 16 µg/mL en cepas aisladas de cloaca y carcasas de pollos ^24^. Los resultados para tetraciclina y eritromicina fueron superiores a lo encontrado en otros estudios que reportaron valores de CIM entre 64 y 128 µg/mL para tetraciclina y eritromicina en cepas de *Campylobacter* spp. aisladas de pollos ^23^^-^^25^. Sadeghi *et al.*^26^ determinaron una CIM de 25 μg/mL para tetraciclina y ciprofloxacina, valores 5 a 6 diluciones por debajo a lo encontrado en el presente trabajo, ellos atribuyen esta baja CIM al uso de antibióticos solo de forma preventiva en la crianza de pollos. En el Perú, Quino *et al.*^4^ reportaron porcentajes de resistencia entre 52% a 100% con una CIM para eritromicina y tetraciclina >128 µg/mL en cepas de *C. coli* aisladas de muestras de humanos y pollos. La CIM para azitromicina encontrada en este trabajo fue más alta en comparación a lo encontrado por Hull *et al.*^25^ quienes reportaron una resistencia de 28% y valores de CIM entre 0,03 y 0,12 µg/mL en cepas a aisladas de carcasas de pollo en Estados Unidos.

Nuestro estudio evidenció un alto porcentaje de cepas *C. coli* aisladas de carne de pollo en Lima Metropolitana con resistencia antimicrobiana. Esto podría estar relacionado con el manejo de las aves durante su crianza donde se usan antibióticos como promotores de crecimiento, se aplican tratamientos con dosificaciones inadecuadas, se usan fármacos vencidos, entre otros ^27^^,^^28^. Esta condición de bacterias resistentes es peligrosa para la salud pública, puesto que estas pueden llegar al humano por manipulación de carne contaminada o por contaminación cruzada ^2^. Otro riesgo es el uso de los mismos grupos de antibióticos en animales y en humanos, la ciprofloxacina es usada para el tratamiento de campilobacteriosis en personas, mientras que la enrofloxacina es el antibiótico de elección en varios programas sanitarios en la crianza de pollos, lo mismo estaría sucediendo con otros grupos de antibióticos que se encuentran disponibles tanto para su uso en animales como para el hombre ^24^. Es claro que los pollos de carne son considerados la fuente primaria de la campilobacteriosis humana y que a pesar que las corporaciones avícolas poseen sistemas de control que aseguran la calidad sanitaria de la carne de pollo, la eliminación total de este microorganismo de la cadena alimentaria es complicada, especialmente por las prácticas de manipulación de carne cruda o la insuficiente cocción a nivel del consumidor final ^29^^,^^30^.

En el Perú existen evidencias que indican que el riesgo de enfermedades gastrointestinales en niños por *Campylobacter* spp. se incrementa cuando conviven con aves de corral (crianza de traspatio) por lo que medidas de contención como corrales y educación sanitaria son sugeridas para reducir la exposición ^31^. Esto es un problema de salud pública, no solo por que involucra la transmisión de la bacteria, también se transmiten los mecanismos de resistencia a antibióticos que poseen. Pollet *et al.*^32^^)^ reportaron un incremento de la resistencia a ciprofloxacina, azitromicina y eritromicina en cepas de *C. jejuni* y *C. coli* aisladas de heces de pacientes con problemas gastrointestinales en hospitales de Lima, Cusco e Iquitos entre el 2001 y 2010, estos autores plantean que el incremento de la resistencia puede ser atribuido a la prescripción frecuente de estos antibióticos en enfermedades infecciosas o a la automedicación; sin embargo, también resaltan que el uso de macrólidos y fluoroquinolonas en la industria animal, especialmente en la avicultura, seria en parte responsable de la resistencia. Por otro lado, Schiaffino *et al.* ^(^^33^ reportaron una alta incidencia de cepas de *Campylobacter* resistentes a quinolonas y macrólidos en heces de niños menores de 2 años de comunidades periféricas a la ciudad de Iquitos. Ellos sugieren que estas bacterias podrían llegar a las personas por la crianza y el sacrificio de aves a nivel doméstico además de la falta de regulación de uso antibióticos en la producción de aves.

Si bien, los tres métodos de sensibilidad antimicrobiana tuvieron una concordancia casi perfecta es importante señalar que la DD solo permitió evaluar resistencia y no determinar la CIM, a diferencia de la ET y MDP. Por otro lado, es evidente que los valores máximos de las concentraciones de los antibióticos en las tiras de ET no son adecuados para la determinación de la CIM en estas cepas. Es por este motivo el uso de la MDP, a pesar de ser un poco más laborioso, es una alternativa adecuada ya que permite preparar *in house* concentraciones que sean necesarias y de esta forma tener una mayor versatilidad dependiendo de las exigencias de las cepas a evaluar. Según Paravissi *et al.*^24^ la comparación de métodos de evaluación es un desafío ya que existe mucha variación en procedimientos, parámetros e interpretación de resultado. Al-Natour *et al*. ^34^ evaluaron la sensibilidad en cepas de *C. jejuni* aisladas de heces de pollos mediante MDP y DD y determinaron que a pesar de que ambas metodologías expresaban resultados concordantes, DD es flexible, conveniente y menos laboriosa y que podría ser utilizada como una prueba rápida inicial. Por otro lado, Azrad *et al.*^35^ determinaron una excelente concordancia entre ET y MDP en la evaluación de la resistencia de eritromicina, ciprofloxacina y tetraciclina en cepas de *C. jejuni* y *C. coli*, resaltando que la MDP tiene la ventaja de que puede ser una metodología automatizada que reduciria el sesgo del operador. Lazou y Chaintoutis ^36^ encontraron concordancias categorizadas como sustancial y casi perfecta entre DD y MDP para los resultados de resistencia de estreptomicina, tetraciclina y ácido nalidíxico en cepas de *C. jejuni* y *C. coli* aisladas de carne ovina y caprina; a pesar de esto los autores resaltan que MDP es un método cuantitativo, lo que es valioso para los clínicos a la hora de decidir un adecuado tratamiento, a diferencia de la DD que solo genera datos cualitativos.

La resistencia a tres o hasta doce antimicrobianos de distintas familias farmacológicas son consideradas cepas multidrogorresistentes (MDR) según lo indicado por Jiménez *et al.*^9^. Nuestros resultados muestran que más del 70% de las cepas fueron resistentes a por lo menos tres antibióticos. Estos porcentajes son altos a diferencia de otros autores que reportaron entre 12,5% a 43,9% ^37^^-^^39^. Algunos países han implementado medidas correctivas y preventivas para la multidrogorresistencia como el uso de alimento sin antibióticos para animales de producción ^40^. En contraposición, el Perú no cuentan con un programa integrado de monitoreo de resistencia para *Campylobacter*, a pesar de que en Sudamérica existen datos que afirman infecciones humanas y se conoce la gravedad de la campilobacteriosis transmitida por alimento como la carne de pollo, aún son escasas las medidas de prevención y control. Los programas de vigilancia ayudarán a identificar el problema, la gravedad y así tomar decisiones e implementar medidas ^28^. Adicionalmente nuestros resultados también deben ser abordados desde el enfoque de “Una salud” ya que la presencia de cepas multidrogorresistentes de *C. coli* en la carne de pollo constituyen un riesgo para que este microorganismo se disemine y afecte no solo a las personas, también a animales de compañía (perros, gatos), animales salvajes (aves), e inclusive a insectos y el agua que cumplen la función de diseminadores; esto podría acelerar la transmisión horizontal de genes de resistencia de *Campylobacter* hacia otros grupos bacterianos como *Enterococcus*, *Staphylococcus* y *Streptococcus.*^41^.

Entre las limitaciones del presente estudio tenemos que para la prueba de ET solo se pudieron evaluar 88 cepas debido a limitaciones en la cantidad de tiras de antibióticos; asimismo, no se pudo evaluar la eritromicina ya que comercialmente estas tiras no están disponibles. Por otro lado, ET es limitado debido a que las concentraciones máximas de las tiras fueron insuficientes para algunas de las cepas evaluadas; en el caso de la DD solo provee datos cualitativos, pero puede ser útil como método de rutina o *screening*.

En conclusión, las cepas de *C. coli* provenientes de carcasas de pollos presentaron un alto porcentaje de resistencia a por lo menos tres antimicrobianos, mientras que para más del 50% de las cepas la CIM fue superior 32 μg/mL para ciprofloxacina, azitromicina, tetraciclina y eritromicina. A pesar que los tres métodos tuvieron concordancia en la evaluación de la resistencia antibacteriana, MDP provee datos cuantitativos y permite modificar las concentraciones utilizadas de acuerdo a la sensibilidad de las cepas, características que son ventajosas sobre los otros dos métodos. Debido a que *C. coli* es una bacteria patógena que puede transmitirse por alimentos de origen animal, es necesario un continuo monitoreo con un enfoque multisectorial que abarque la salud humana, animal y el medio ambiente, no solo de su presencia, también de la resistencia antimicrobiana, determinar la CIM y sobre todo realizar una evaluación con ayuda de la biología molecular de los genes de resistencia que están presentes en estas cepas.
